# Genetically diverse CC-founder mouse strains replicate the human influenza gene expression signature

**DOI:** 10.1038/srep26437

**Published:** 2016-05-19

**Authors:** Husni Elbahesh, Klaus Schughart

**Affiliations:** 1 University of Tennessee Health Science Center, Memphis, Tennessee, United States of America; 2 Department of Infection Genetics, Helmholtz Centre for Infection Research, Braunschweig,Germany; 3 University of Veterinary Medicine Hannover, Hannover, Germany

## Abstract

Influenza A viruses (IAV) are zoonotic pathogens that pose a major threat to human and animal health. Influenza virus disease severity is influenced by viral virulence factors as well as individual differences in host response. We analyzed gene expression changes in the blood of infected mice using a previously defined set of signature genes that was derived from changes in the blood transcriptome of IAV-infected human volunteers. We found that the human signature was reproduced well in the founder strains of the Collaborative Cross (CC) mice, thus demonstrating the relevance and importance of mouse experimental model systems for studying human influenza disease.

Recent studies comparing inflammatory responses in mice and humans have reported conflicting results regarding whether or not the transcriptome signature found in human patients can be replicated in mice^1^,^2^. In addition, the study itself was limited by the use of a single inbred mouse strain, C57BL/6J, and not investigating responses to acute infections. Another study investigated changes in blood transcriptomes of human volunteers following H1N1 and H3N2 human influenza A virus (IAV) infection and identified a gene expression signature that distinguished non-infected from infected conditions^3^. The specificity and sensitivity of this signature was subsequently confirmed using another cohort^4^. Therefore, we studied transcriptomic changes following IAV infections in the founder strains of a genetically highly diverse experimental mouse model, the Collaborative Cross (CC)^5^. Using the founder strains of the CC, we aimed to address two fundamental questions: How well does the mouse reproduce the human gene signature? How much does genetic diversity influence the profile of this gene signature?

## Results

### Gene expression in the blood of infected mice reproduces human signature profile

**Figure 1:**
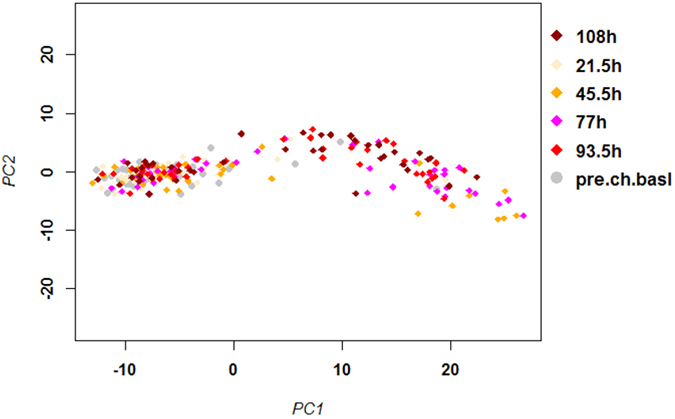
PCA analysis of signature gene expression values in the blood of human volunteers. Principle component analysis (PCA) was performed with normalized log_2_–transformed expression values for the signature genes identified in infected human volunteers (omitting *XIST*)^3^. Horizontal and vertical axis represent principle component 1 and 2, respectively. The first two principal components represent 85% and 5%, respectively, of the total variation. Dots represent individual samples from patients at the indicated times p.i. Colors represent the non-infected samples (pre.ch.basal) and samples from different time points p.i. as indicated.

The similarity in overall gene expression changes following IAV infection in humans and mice can be best appreciated by a principal component analysis (PCA). Consistent with previous reports^3^, we found a separation in infected and control samples from infected human volunteers, in particular at later time points (after 77 hpi) for the 55 (unique genes from Table S1) IAV signature genes (Fig. 1). It should be noted that in our study we did not include the XIST gene, which was part of the original signature gene list (see M&M for details). Several IAV-infected samples grouped with the baseline non-infected controls and did not show an IAV-specific response even at the later times p.i. The phenomenon where infected/treated subjects do not show clearly distinct responses is often observed in human studies. A possible explanation for this effect is most likely the influence of additional environmental factors, life style, diet, health conditions and infection dose; none of which can be well-controlled in human studies.

**Figure 2:**
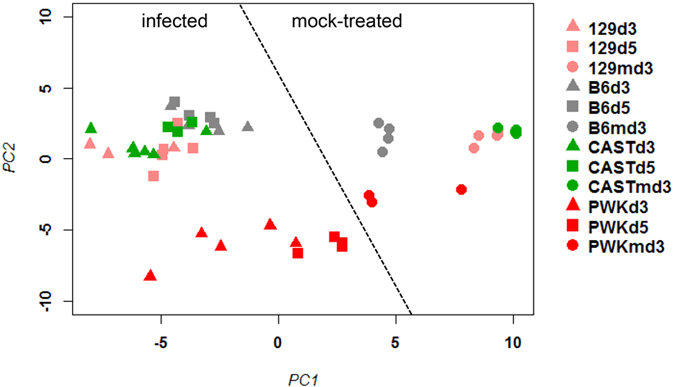
PCA analysis of gene signature expression values in the blood of CC founder mice. Principle component analysis (PCA) was performed with normalized log_2_–transformed gene expression values for the mouse orthologues (Table S2) of the human signature genes (omitting *XIST*) after IAV infection with 10 FFU H3N2 of mice from four CC founder strains^6^. Horizontal and vertical axis represent principle component 1 and 2, respectively. The first two principal components represent 54% and 17%, respectively, of the total variation. Dots represent individual samples from controls at day 3 post treatments (labelled md3) or infected mice. Colors represent the different mouse strains. Symbols represent controls and different times p.i.

Next, we analyzed changes in gene expression for the human signature genes in the peripheral blood transcriptome of four CC founder strains (C57BL/6J, 129S1/SvlmJ, CAST/EiJ, PWK/PhJ) infected with mouse adapted H3N2 IAV that were published previously by our laboratory^6^. Forty-five of the human signature genes were represented on the array that was used for the analysis (Table S2). The PCA for these 45 signature genes showed a clear separation of mock-infected control and IAV-infected mice (Fig. 2) in the peripheral blood. It is noteworthy that this separation between infected and non-infected mouse samples was much more distinct compared to the human samples (Fig. 2). Furthermore, also infected PWK/PhJ mice that exhibit very mild symptoms after infection^6^ were well separated from the non-infected PWK/PhJ control mice^6^. These results demonstrate that the CC mouse-model can faithfully reproduce the human IAV gene signature in the blood. Therefore, our data validates the mouse IAV-model system as a well-controlled experimental tool for studying human IAV infections.

### Analysis of single gene expression changes distinguishes high and low responder individuals

**Figure 3:**
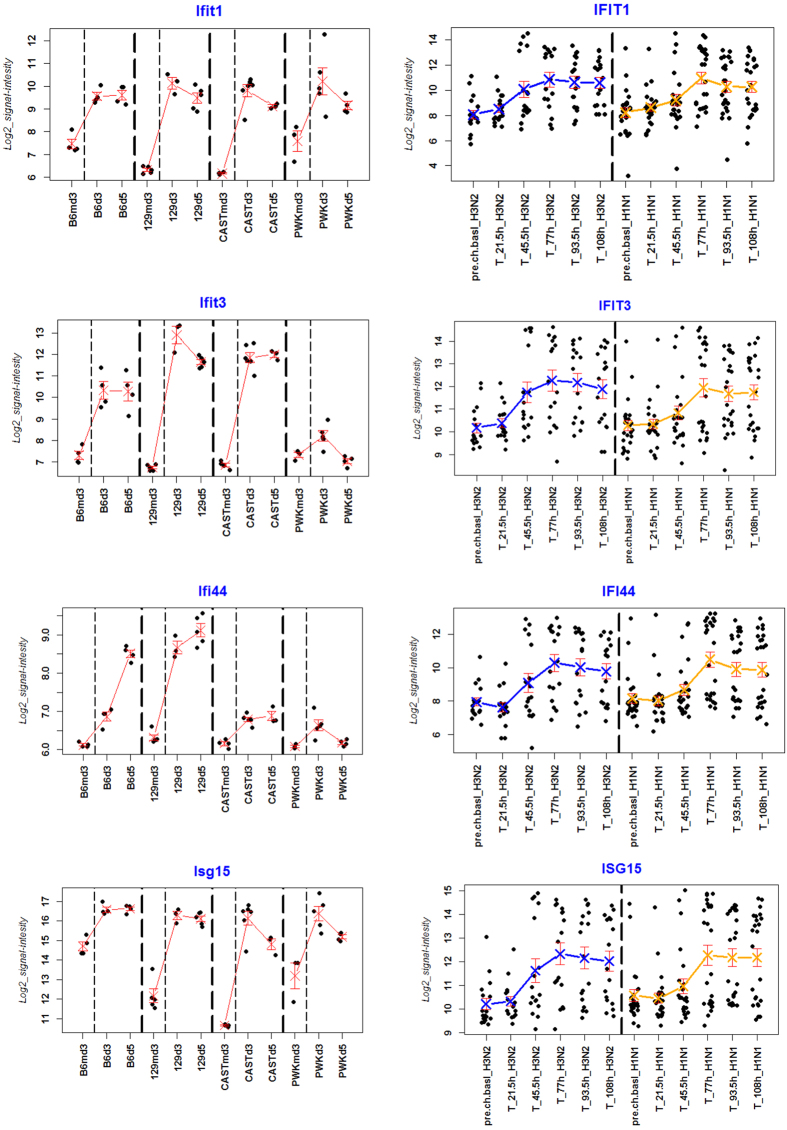
Single gene expression values in the blood of mice and human patients for selected genes. Normalized log_2_–transformed relative gene expression values of *IFIT1, IFIT3, IFI44* and *ISG15* in mice (left panel) and humans (right panel). Human data were taken from^3^, mice data from^6^. Dots represent values from individual mice and human volunteers. Crosses represent mean expression values per group, bars represent +/− 1 SEM (standard error of mean). Mock-infected control mice are designated ‘md3’, infected mouse groups are labelled ‘d3’ and ‘d5’ representing the time points p.i. Mouse strains: B6: C57BL/6J; 129: 129S1/SvlmJ; CAST: CAST/EiJ; PWK: PWK/PhJ. For human samples, pre.chal.basel’ represents control samples of volunteers before infection and ‘T’ designates samples from infected patients at the indicated time points. Infections were performed in humans with two IAV subtypes, H3N2 and H1N1.

To compare gene expression patterns between human and mouse blood samples for the IAV signature profile in more details, we selected the ten most highly-expressed genes in humans. We compared the mean expression values for these genes in mice and humans (Fig. S1). As an example, expression levels in individual mice and human subjects from four genes are shown in Fig. 3. Although mean values clearly distinguished IAV-infected patients from uninfected controls at later times of infection, a large variation between individual human samples was observed (Fig. 3). In contrast to the individual variation in the human data set, the individual samples in the mouse data set consistently and reproducibly showed similar values at all time points between mice from the same inbred mouse strain. However, strong differences could be observed between individuals from different mouse strains. For example, highly susceptible strains (C57BL/6J, 129S1/SvlmJ, CAST/EiJ) exhibited a strong increase in *IFIT3* and *IFIT44* expression after infection but only a relatively low increase in expression was observed in the resistant strain PWK/PhJ (Fig. 3). We and others have previously reported that the magnitude of the inflammatory response following IAV infection is largely correlated with viral load as well as genetic background of the mouse-strains examined (reviewed in^7^). These findings suggest that genetic diversity is a major contributor of the variation in response to infection between individual humans.

## Discussion

In this study, a comparison of IAV gene signature profiles clearly demonstrated that CC founder mice faithfully reproduced the human IAV-signature highlighting them as an ideal model to study human host responses to IAV infections. Although our findings are in contrast to results described by Seok *et al*.^1^, who did not find similar responses in mice and humans associated with inflammatory diseases, our findings are supported by the analysis performed by Takao *et al*.^2^. Most importantly, our study does not rely on a single mouse strain but rather uses multiple CC founder strains. The approach in our study provides a more dynamic response range that can accommodate the genotypic differences among individuals of a given population.

The PCA analysis for mice and human transcriptome changes revealed a larger variation explained by PC2 in mice than in humans. PC2 in mice separated the different mouse strains according to their susceptibility to infection: C57BL/6J, 129S1/SvlmJ, and CAST/EiJ are highly susceptible whereas PWK/PhJ is very resistant^6^. We previously reported that the phenotypic responses after IAV infection of CC founder mice, such as body weight loss and survival, are highly heritable^6^. Because a different mouse strain means a different genetic background, the variation observed for PC2 in mice can be interpreted as an effect that is mainly due to genetic differences. Accordingly, we hypothesize that genetic diversity in humans will also significantly influence the IAV-signature, especially if human patients from different ethnic backgrounds were studied. Therefore, it is important to also consider genetic diversity in future patient studies to provide a more detailed and comprehensive explanation of factors that drive variation in responses to infections in humans.

By evaluating the observed variables (genes) as vectors in a PCA biplot, the magnitude and polarity of each variable’s contribution to the first two principal components can be visualized (data not shown). It revealed that both mouse and human responses along PC1 (the infection response) are largely driven by interferon-response genes (e.g. *ISG15, IFIT1*). In the human data set, all genes were contributing to PC1 (the polarity of infection response) whereas in the mouse, several genes were also contributing to PC2. We attribute this to the fact that the mouse strains exhibited a high level of genetic diversity whereas the genetic diversity of the human volunteers was fairly homogeneous and composed mainly of Caucasian background (37 Caucasians, one Hispanic, one African American, one Native American, one mixed)^3^. Thus, we conclude that our results were less driven by species-differences than the genetic make-up of the populations that were studied. This hypothesis can be tested in future human studies using cohorts that include participants of other ethnic origin and then compare the results to the profiles from a larger strain collection of CC mice. Because the genetic architecture of the CC population (with respect to single nucleotide polymorphisms, structural variants, indels) is different from human populations, we may expect some plasticity in the up/down-regulation of single genes in mice. However, it is expected that the same pathways (i.e. interferon pathway, endocytic pathway, etc) and even the same arms of those pathways would be identical to those up/down-regulated in humans. Thus, genes that are regulated in the same fashion in the CC mouse population and a given human cohort may represent genes that are also robustly regulated in genetically diverse human populations. Therefore, studies identifying expression profiles commonly associated with susceptibility and resistance in both species are of great value.

A major weakness in carrying out human studies is typically due to cohorts being too small to sufficiently provide coverage of human genetic variability. This same weakness is also observed in other model systems using outbred and larger size experimental animals such as ferrets and swine. The CC mouse genetic reference mouse-model specifically addresses these limitations. The CC founder mice gave rise to the CC genetic reference population with hundreds of strains in which the parental genomes are segregating^5^. The ability to carry out well-controlled experiments using multiple mouse strains makes mice an indispensable model system for understanding and profiling responses to IAV infections in humans. Coupled with the large genetic heterogeneity of the CC founder strains and their derivative CC strains, mice will be essential to understand the varying responses to infections between individual humans, the contribution of genetic variation on the host response and the molecular mechanism that regulate these responses.

## Material and Methods

### Ethics statement

All experiments in mice were carried out in accordance with the approved guidelines. The protocols were approved by an external committee according to the national guidelines of the animal welfare law in Germany (BGBl. I S. 1206, 1313 and BGBl. I S. 1934). The protocol used in these experiments has been reviewed by an ethics committee and approved by the ‘Niedersächsisches Landesamt für Verbraucherschutz und Lebensmittelsicherheit, Oldenburg, Germany’ (Permit Numbers: 33.9.42502-04-051/09 and 3392 42502-04-13/1234).

### Human and mouse transcriptome data sets

As data set for the human blood transcriptomes, we used gene expression data from the blood of human volunteers which had been infected with H1N1 and H3N2 IAV by Woods *et al*.^3^. The data set was downloaded from GEO (http://www.ncbi.nlm.nih.gov/geo/; GSE52428). For better visibility of the larger data set, we did not represent all groups but selected the most informative ones: one control group (control ‘pre.chal.basel’), an early time point (21.5 hr p.i.), a median (45.5 hr) and three late time points (77, 93.5 and 108 hr p.i., respectively). The later time points represent the stages after infection where IAV signature genes best distinguished infected patients from controls^3^.

The human influenza signature gene list has been taken from Table S4 from^3^. This list (Table S1) contains the top 50 genes from the discriminative factors that were obtained after infection of volunteers with H1N1 and H3N2 challenge trials and been ranked by order of individual contribution to the strength of the factor^3^. We have generated a combined unique gene list from this table that encompasses 55 genes in total.

A first analysis of the human gene expression profiles for the signature genes of the data set from Woods *et al*.^3^ revealed that male and female volunteers were represented as two separate groups along PC2 (Fig. S2). This was due to the inclusion of XIST in the signature gene list, a gene only expressed in females. We thus removed XIST from the gene list for further analyses.

The transcriptome data from the blood of four CC founder mice^5^, C57BL/6J, 129S1/SvlmJ, CAST/EiJ, and PWK/PhJ, has been described in^6^. Briefly, mice were infected with 10 FFU H3N2 mouse-adapted influenza A virus and blood was prepared at day 3 and day 5 p.i. As controls, mice were mock infected and blood was collected at day 3 post treatments. Whole transcriptome analysis was performed on Agilent’s mouse 4 × 44 k microarrays arrays as described^6^. The data set has been deposited at GEO (http://www.ncbi.nlm.nih.gov/geo/; GSE74077).

### Gene expression analysis

Array data were analyzed using the R software package^8^. Pre-processing steps of mouse array data included background correction, quantile normalization and annotation using the MmAgilentDesign026655.db^9^, limma^10^, and Agi4 × 44PreProcess^11^ packages. Array data from human infections were downloaded from GEO (http://www.ncbi.nlm.nih.gov/geo/; GSE52428), log_2_ transformed and quantile normalized before further analysis. Principal component analysis (PCA) was performed using the affycoretools package^12^.

## Additional Information

**How to cite this article**: Elbahesh, H. and Schughart, K. Genetically diverse CC-founder mouse strains replicate the human influenza gene expression signature. *Sci. Rep.* **6**, 26437; doi: 10.1038/srep26437 (2016).

## Supplementary Material

Supplementary Information

Supplementary Information

Supplementary Information
