# Supplementary material for: Genetically diverse CC-founder mouse strains replicate the human influenza gene expression signature
Source: Sci Rep. 2016 May 19;6:26437. doi: 10.1038/srep26437 (PMC4872221; doi:10.1038/srep26437)
Supplement: Supplementary Information [file srep26437-s1.pdf]

Supplemental material

Genetically diverse CC-founder mouse strains replicate the human influenza gene expression signature profile

Author affiliations

Husni Elbahesh[1] and Klaus Schughart[1,2,3,4]

[1]*University of Tennessee Health Science Center, Memphis, Tennessee, United States of America*

[2]*Department of Infection Genetics, Helmholtz Centre for Infection Research, Braunschweig*

[3]*University of Veterinary Medicine Hannover, Hannover, Germany*

[4]Corresponding author at: Helmholtz Centre for Infection Research, Dept. of Infection Genetics, Inhoffenstr.7, D-38124 Braunschweig

E-mail address: klaus.schughart@helmholtz-hzi.de

Supplemental Figures

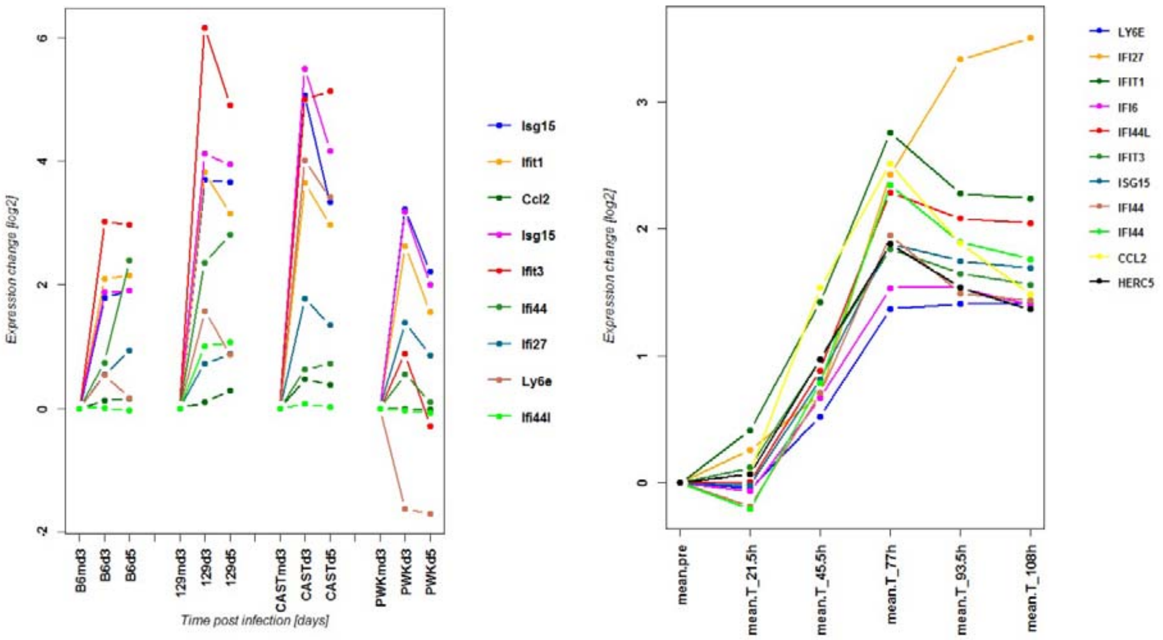

Figure S1: Changes in the expression levels of top 10 signature genes in mice and humans

Left panel: Expression values of normalized log_2 transformed signal intensities in the blood of H1N1 infected mice at different time points p.i. relative to expression levels in mock-infected control mice. Please note that less than 10 genes are shown for mice because not all human genes had orthologues represented on the mouse expression array. Mock-infected control mice are designated ‘md3’, infected mouse groups are labelled ‘d3’ and ‘d5’ representing the time points p.i. Mouse strains: B6: C57BL/6J; 129: 129S1/SvImJ; CAST: CAST/EiJ; PWK: PWK/PhJ. Right panel: Expression values of normalized log_2 transformed signal intensities in the blood of infected human volunteers at different time points p.i. relative to expression levels in control samples before infection (pre.chal.basel’). ‘T’ designates samples from infected patients at the indicated time points. Infections were performed in humans with two IAV substrains, H1N1 and H3N2.

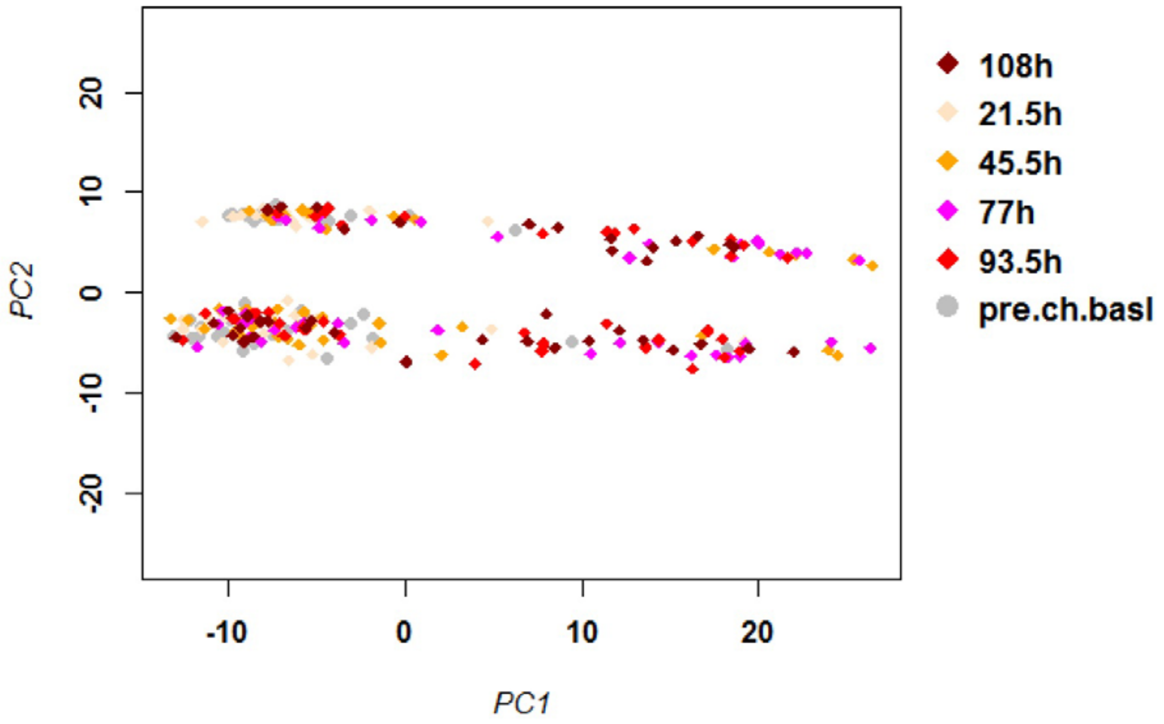

Figure S2: PCA analysis of signature gene expression values in the blood of human volunteers

Principle component analysis (PCA) was performed with normalized $\log_2$–transformed expression values for the signature genes identified in infected human volunteers (including *XIST*)[1]. Horizontal and vertical axis represent principle component 1 and 2, respectively. The first two principal components represent 71 % and 16 %, respectively, of the total variation. Dots represent individual samples from patients at the indicated times p.i. Colors represent the non-infected samples and samples from different time points p.i. Note the two distinct groups (males and females) separated by PC2 which is due to inclusion of *XIST* as a signature gene.

Supplemental Tables

Table S1: Signature gene list from table S4 from[1]*.*

Table S2: Mouse signature genes that were identical by name to the human signature genes (Table S1) and represented on the microarray.

References

1 Woods, C. W. *et al.* A host transcriptional signature for presymptomatic detection of infection in humans exposed to influenza H1N1 or H3N2. *PLoS One* **8**, e52198, doi:10.1371/journal.pone.0052198 (2013).
